# Chemopreventive Effects of Lycopene in miR‐21 Knockout and Wild‐Type Pancreatic Cancer Cells

**DOI:** 10.1155/sci5/8865270

**Published:** 2026-02-12

**Authors:** Basak Dalbayrak, Burcu Taluğ Taştan, Tuğçe Temel, Pinar Obakan Yerlikaya, Pinar Uysal Onganer, Elif Damla Arisan

**Affiliations:** ^1^ Institute of Biotechnology, Gebze Technical University, Gebze, 41400, Kocaeli, Türkiye, gyte.edu.tr; ^2^ Department of Molecular Biology and Genetics, Graduate School of Natural and Applied Sciences, Gebze Technical University, Gebze, 41400, Kocaeli, Türkiye, gyte.edu.tr; ^3^ Department of Molecular Biology and Genetics, Istanbul Medeniyet University, Istanbul, 34700, Türkiye, medeniyet.edu.tr; ^4^ Cancer Mechanisms and Biomarkers Research Group, School of Life Sciences, University of Westminster, London, W1W 6UW, UK, westminster.ac.uk

## Abstract

Pancreatic cancer is one of the most lethal malignancies, characterized by late diagnosis, rapid progression, and resistance to conventional therapies. The oncogenic microRNA miR‐21 is frequently upregulated in pancreatic tumors and contributes to tumor growth, migration, and chemoresistance by targeting tumor suppressor genes. Lycopene, a naturally occurring carotenoid with antioxidant and anti‐inflammatory properties, has shown promise as a chemopreventive agent in several cancer types. This study investigates the therapeutic potential of lycopene in human pancreatic cancer cell lines (PANC‐1 and MIA PaCa‐2) and their miR‐21 knockout counterparts. Treatment with 50 μM lycopene significantly reduced cell viability, colony formation, migration, and spheroid integrity and decreased intracellular reactive oxygen species (ROS) levels, with more pronounced effects in miR‐21‐deficient cells. These findings highlight the role of miR‐21 in modulating lycopene sensitivity and support the potential of lycopene as an adjunctive therapeutic agent in pancreatic cancer.

## 1. Introduction

Pancreatic cancer remains one of the most lethal malignancies worldwide, with a 5‐year survival rate below 10% and an increasing incidence of 0.5%–1% annually. Pancreatic cancer remains fatal due to late detection and aggressivity of the tumor type. The cold tumor generally has a low mutational burden and exhibits inadequate T‐cell filtering [1]. These characteristics make it challenging to target with smart drugs and weaken the immune defense against the lesions. The tumor either becomes extremely large or has already metastasized by the time the cancer is discovered, as in the clinic. Unlike breast or uterine cancer, there are no effective surveillance methods for pancreatic cancer, making detecting and treating this disease much more difficult. Pancreatic cancer will be the second leading cause of cancer‐related death by 2030 [2].

In recent years, microRNAs (miRNAs) have emerged as key regulators of tumor biology. These small, noncoding RNAs (∼22 nucleotides) modulate gene expression posttranscriptionally and are involved in processes such as cell proliferation, apoptosis, migration, and drug resistance [3–5]. Among these, miR‐21 is one of the earliest and most extensively studied oncomiRs. It promotes tumor progression primarily by targeting tumor suppressor genes such as phosphatase and tensin homolog (PTEN), Programmed cell death protein 4 (PDCD4), and SPRY2, thereby enhancing proliferation and survival [6–8]. Overexpression of miR‐21 has been reported in various cancers and is associated with poor survival outcomes [9, 10]. miR‐21 modulates pathways related to proliferation, migration, angiogenesis, and apoptosis resistance, and its role as both an oncomiR and a potential biomarker has been widely documented [11]. For example, Koga and colleagues reported that miR‐21 and miR‐92a were significantly elevated in colorectal cancer patients compared to controls [12]. Elevated miR‐21 expression has also been observed in several other cancer types, including breast, gastric, colon, lung, pancreatic, and ovarian malignancies [13]. In lung cancer, miR‐21 was shown to regulate cell growth and the epithelial–mesenchymal transition (EMT) process through the PTEN/Akt/GSK3β signaling pathway [14]. In addition, in pancreatic ductal adenocarcinoma (PDAC) cell lines such as PANC‐1 and MIA PaCa‐2, miR‐21 expression has been linked to enhanced cancer stem‐like characteristics [15].

Lycopene is a red carotenoid found in ripe tomatoes, grapefruits, and red watermelons, known for its antioxidant, anti‐inflammatory, and chemopreventive properties. Its bioavailability increases in the presence of dietary fats and with heat processing. Lycopene’s extended conjugated double bonds enable it to function as an effective singlet oxygen scavenger and free radical quencher. These properties have been linked to its anticancer potential across multiple malignancies. For instance, lycopene has been shown to inhibit proliferation and induce apoptosis in gastric and colon cancer cells without affecting normal cells [16, 17]. In addition, a Phase I clinical trial in metastatic prostate cancer patients reported that lycopene combined with docetaxel improved drug pharmacokinetics and modulated relevant oncogenic biomarkers, supporting its potential as a safe adjuvant therapy [18]. Furthermore, clinical studies have demonstrated that dietary lycopene supplementation increases tissue levels and reduces oxidative DNA damage in cancer patients [19, 20].

In pancreatic cancer, lycopene has been shown to inhibit nuclear factor κ‐light–chain enhancer of activated B cell (NF‐κB)–mediated reactive oxygen species (ROS) production and promote apoptosis in vitro [21]. In addition, it downregulates AKT2 expression and upregulates the tumor‐suppressive miR‐let‐7f‐1, leading to reduced proliferation and increased apoptosis in prostate cancer cells [22]. These findings collectively suggest lycopene’s multitargeted therapeutic potential and support further investigation in miR‐21‐modulated pancreatic cancer models.

Given the established roles of miR‐21 in promoting oncogenesis and therapy resistance, the multitargeted biological actions of lycopene, investigating their potential interaction, present a compelling therapeutic opportunity. This study was designed to evaluate the chemopreventive effects of lycopene on miR‐21 knockout and wild‐type pancreatic cancer cells (PANC‐1 and MIA PaCa‐2). By focusing on this interplay, the research aims to provide mechanistic insight into whether suppression of miR‐21 enhances the antitumor efficacy of lycopene, and whether lycopene may hold value as an adjunct to current therapeutic strategies in pancreatic cancer.

## 2. Materials and Methods

### 2.1. Cell Culture

Human pancreatic cancer cell lines with PANC‐1 and MIA PaCa‐2 cells were from the American Type Culture Collection (ATCC) (LGC Promochem, Rockville, MD). miR‐21 KO cells, PANC‐1 miR‐21 KO_4_, and MIAPaCa‐2 miR‐21 KO_2_ were provided by Pınar Uysal Onganer from the University of Westminster. Cells were incubated to 80% confluence in 25 cm^2^ flasks in Dulbecco’s modified Eagle’s medium (GIBCO‐Life Technologies, Carlsbad, CA, USA) containing 10% fetal bovine serum (Pan–Biotech GmbH, Aidenbach, Germany) and 1% penicillin/streptomycin (Pan–Biotech GmbH, Aidenbach, Germany) at 37°C in a humidified incubator with 5% CO_2_ according to ATCC recommendations.

### 2.2. Cell Viability Assay

Cell viability was assessed using the 3‐(4,5‐dimethylthiazol‐2‐yl)‐2,5‐diphenyltetrazolium bromide (MTT) assay, as previously described [23]. The effects of lycopene on cell viability were determined using the colorimetric MTT assay. Cells (1 × 10^4^ per well) were seeded into 96‐well plates and incubated overnight. Lycopene (50 μM, Sigma‐Aldrich, USA) was applied for 24 h and 48 h. At the end of the treatment period, 10 μL MTT reagent (5 mg/mL; Sigma, St Louis, MO, USA) was added to the cell culture medium for 4 h. Formazan crystals formed by mitochondrial reactions were dissolved using 100 μL DMSO (TEKKIM, Turkey). Then, the absorbance of each suspension was determined at 570 nm using a microplate reader (VarioSkan, Thermo Fisher, USA).

### 2.3. Colony Formation Assay

To evaluate long‐term proliferative capacity, a standard clonogenic assay was performed [24]. Cells were seeded at 2.5 × 10^3^/well in complete media in 6‐well plates and allowed to adhere overnight. 50 μM lycopene was added to the appropriate wells after 72–48–24 h to determine the time‐dependent lycopene effect. At the end of the targeted exposure time, the lycopene‐containing medium was replaced with a fresh medium, and the cells were allowed to form colonies in the complete medium for 10–14 days. Then, the medium was removed and washed with 1000 μL 1x PBS. Colonies were then fixed with a solution of acetic acid and methanol (1:3) for 10 min and stained with 0.5% crystal violet for 5 min.

### 2.4. Fluorescent Microscopy

Cells (2.5 × 10^4^ per well) were seeded in a 24‐well plate and incubated overnight. The next day, 50 μM lycopene was applied for 48 h, and the following day, 50 μM lycopene was applied for the 24 h group. After lycopene application, the lycopene‐containing medium was removed, and the cells were washed with 1x PBS to remove FBS. Then, dyes (4 nM 3,3′‐dihexyloxacarbocyanine iodide [DiOC6], 0.5 μg/mL propidium iodide [PI], 10 μg/mL 2′,7′‐dichlorofluorescein diacetate [DCFDA/H2DCFDA], 1 μM MitoTracker, and 10 μg/mL 4′,6‐diamidino‐2‐phenylindole [DAPI]) were diluted in serum‐free DMEM and applied to the cells. Microscopic images were taken using appropriate wavelengths (ZOE, Bio‐Rad).

### 2.5. Wound‐Healing Assay

Wound‐healing assay was assessed as previously described [24]. Cells were seeded in 24‐well plates at a density of 2 × 10^5^ cells/well and grown to ∼90% confluence. A uniform scratch was introduced using a sterile 10 μL pipette tip. After washing with PBS, cells were incubated in complete DMEM (control) or DMEM containing 50 μM lycopene. Cells were incubated at 37°C in a CO_2_ incubator for 48 h. Wound closure was imaged at 0 h, 24 h, and 48 h using a light microscope (Olympus CKX53). The wound gap was measured using ImageJ software, and the relative wound closure percentage was calculated at 48 h as: ([initial width − final width]/[initial width] × 100) and presented as bar graphics using GraphPad software (Version 9.5.1). Data represent triplicates.

### 2.6. Hanging Drop Assay

Three‐dimensional spheroid cultures were prepared using the hanging drop method as previously described [24]. PANC‐1 and MIA PaCa‐2 cells (2.5 × 10^3^ per 10 μL drop) were suspended in either control or lycopene‐supplemented (50 μM) complete DMEM and pipetted onto the inner side of 60 mm petri dish lids. 50 droplets were seeded in total for each plate. The lid of the plate was gently flipped upside down. The base was filled with 3‐mL PBS to maintain humidity. Cells were incubated at 37°C in a CO_2_ incubator. Spheroids were allowed to form for over 72 h. Imaging was performed every 24 h, and 10 spheroids per group were analyzed using ImageJ for diameter measurements. At 72 h, spheroids were stained with DiOC6 as a viability marker and PI as a necrosis marker to assess viability and observed under fluorescence microscopy (ZOE Fluorescent Cell Imager, Bio‐Rad Laboratories).

### 2.7. Statistical Analysis

Statistical analyses were performed using GraphPad Prism 9.2.0. All experiments were conducted in at least three biological replicates. Data are presented as mean ± standard deviation (SD). Statistical significance was evaluated using two‐way ANOVA followed by Tukey’s or Bonferroni’s multiple comparison test. *p* values < 0.05 were considered statistically significant with a 95% confidence interval. Results without explicit statistical markers indicate nonsignificant differences (*p* > 0.05). Image analysis (e.g., colony size, wound closure, and fluorescence intensity) was performed using ImageJ software (NIH).

## 3. Results

MTT test results were analyzed via two‐way ANOVA and Tukey’s multiple comparison test. The results for PANC‐1 and PANC‐1 miR‐21 KO_4_ indicated that the cell viability decreased significantly after 48 h of 50 μM lycopene treatment (Figures 1(a), 1(b)). Lycopene treatment for MIA PaCa‐2 caused a significant decrease after 48 h; while the lycopene treatment significantly reduced the cell viability of MIA PaCa‐2 miR‐21 KO_2_ after 24 h application (Figures 1(c) and 1(d)). The colony formation of these four cell lines showed similar results as the MTT assay (Figure 1(e)).

Figure 1MTT assays and the colony formations for (a) PANC‐1, (b) PANC‐1 miR‐21 KO_4_, (c) MIAPaCa‐2, and (d) MIAPaCa‐2 miR‐21 KO_2_, with time‐dependent lycopene (50 μM) treatment applied. The error bars represent the standard deviations of 3 biological replicates. (e) The colony formation assay was achieved for all cell lines. The two‐way ANOVA test was applied with Tukey’s comparison test.  ^∗^ *p* < 0.05, ^∗∗^ *p* < 0.01, ^∗∗∗^ *p* = 0.0002, and ^∗∗∗∗^ *p* < 0.0001.

**Figure:**
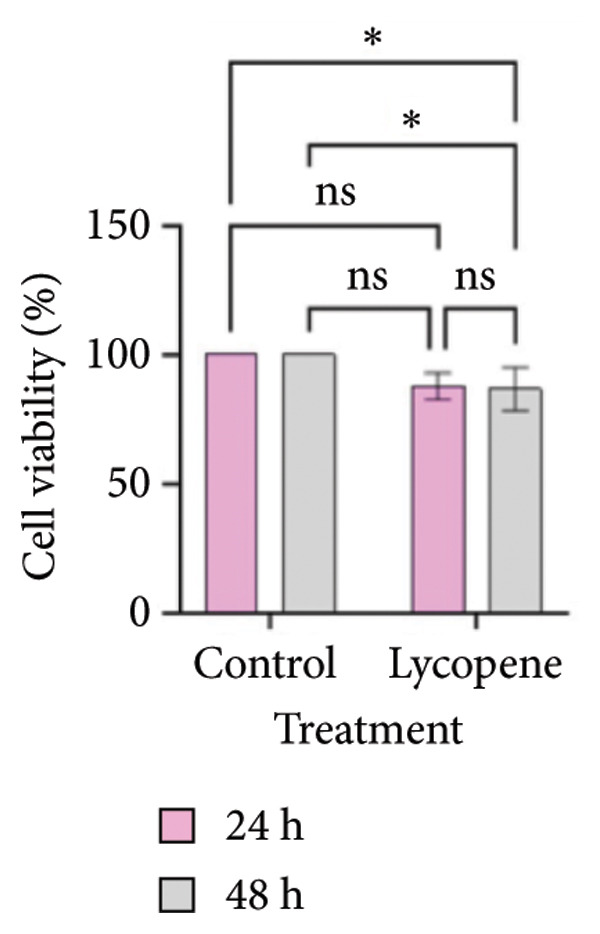
(a)

**Figure:**
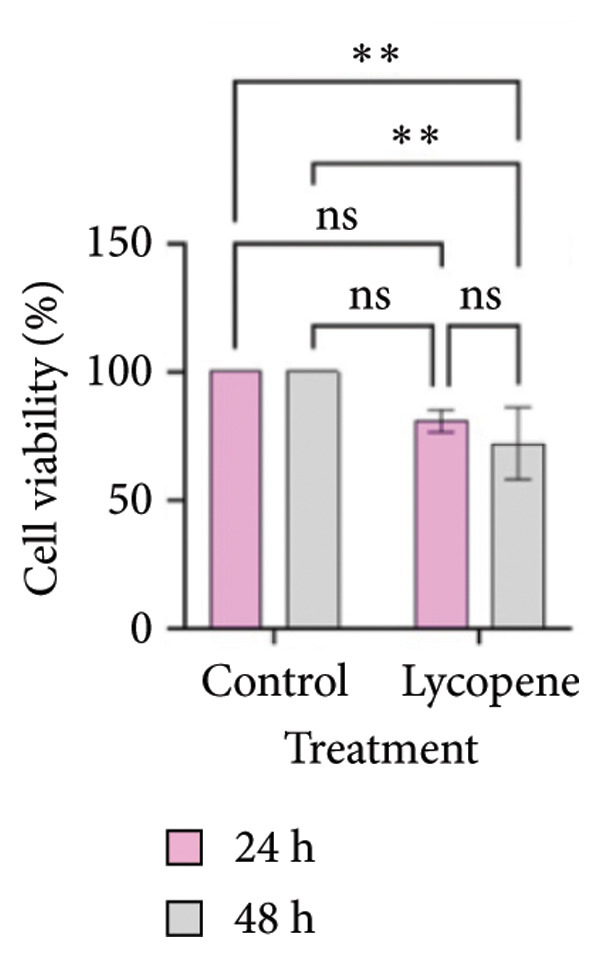
(b)

**Figure:**
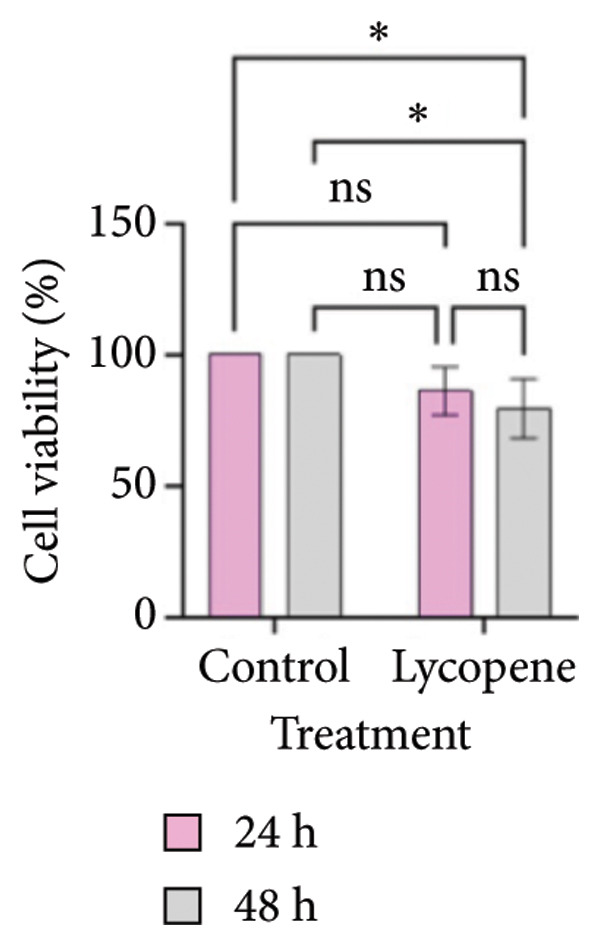
(c)

**Figure:**
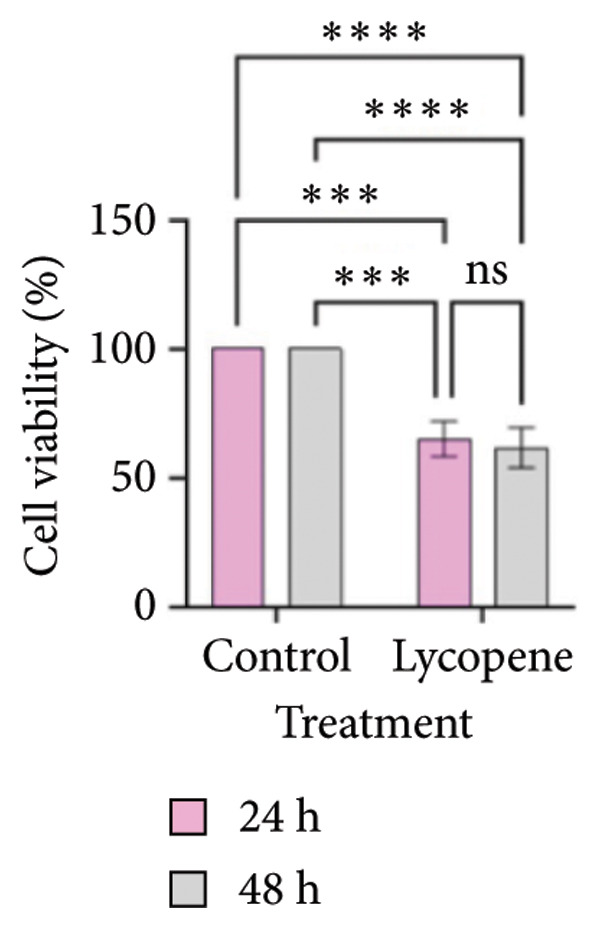
(d)

**Figure:**
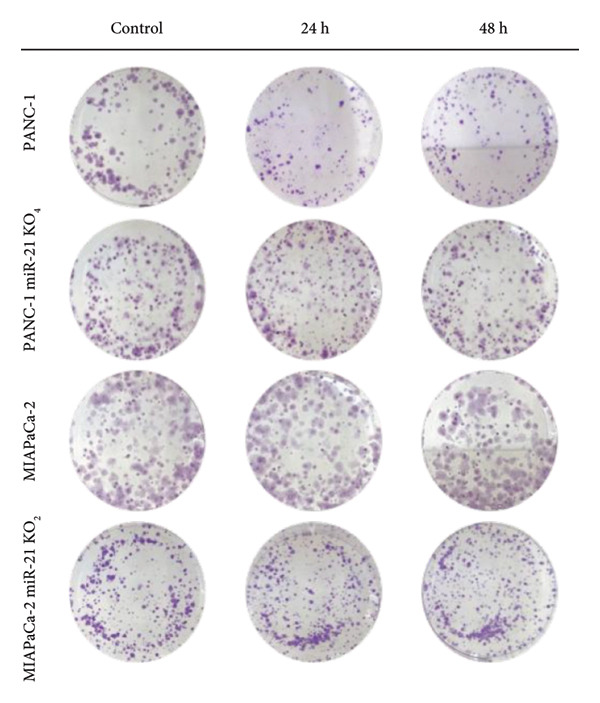
(e)

The cellular effects of 50 μM lycopene application in four cell lines were visualized with fluorescent microscopy using various dyes (Figures 2 and 3). Cells were evaluated with the DAPI dye, which binds to DNA. DiOC6 has been used to assess cellular viability by detecting cells with healthy mitochondrial activity. MitoTracker dye was used for the mitochondrial activity such as DiOC6. PI stain was applied to investigate the treatment‐dependent cytotoxicity of lycopene by staining dead cells. DCFDA/H2DCFDA dye was used only to detect reactive oxidative stress. Considering fluorescence images for both PANC‐1 and PANC‐1 KO_4_ cells, there was a decrease in living cells with increasing dead cell numbers (Figures 2(a) and 2(b)). The dead/live cell ratio of MIA PaCa‐2 and MIA PaCa‐2 KO_2_ increased after lycopene application (Figures 2(c), 2(d). After lycopene treatment for all four cell lines, the ROS activation decreased, while there were no differences in the nontreated control groups (Figure 3).

Figure 2Fluorescence images of (a) PANC‐1, (b) PANC‐1 miR‐21 KO_4_, (c) MIAPaCa‐2, and (d) MIAPaCa‐2 miR‐21 KO_2_. Living cells were stained with DiOC6 (green), dead cells were indicated with PI (red), cell nuclei were identified with DAPI (blue), and mitochondria were indicated with MitoTracker (red). The treatment groups were 50 μM lycopene application for 24 h. The scale bars of the images were 100 μm.

**Figure:**
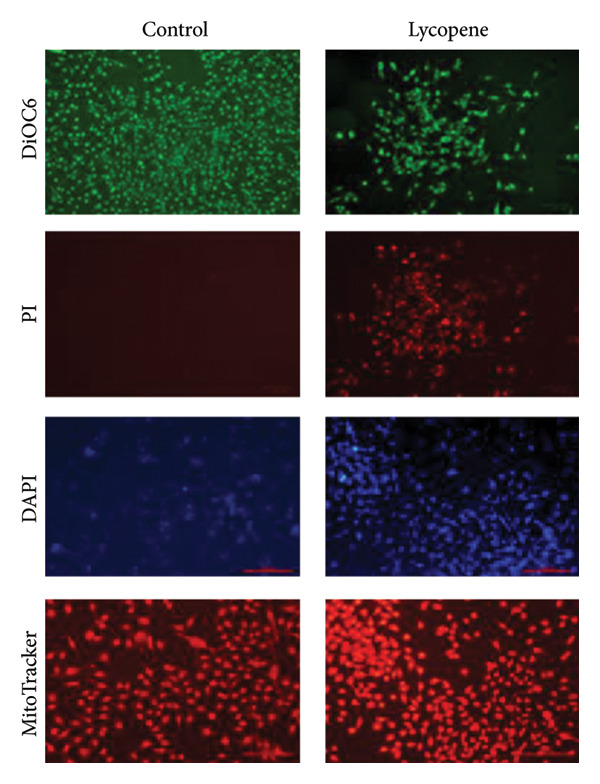
(a)

**Figure:**
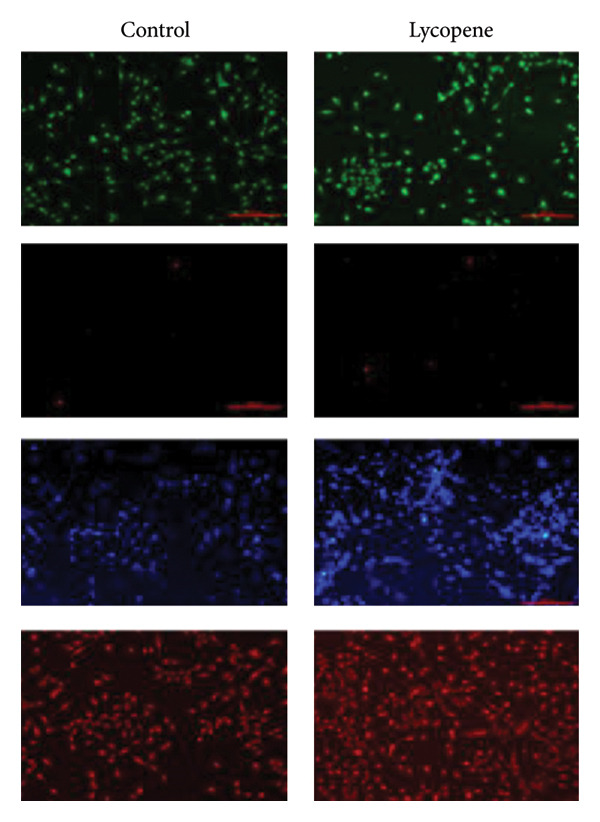
(b)

**Figure:**
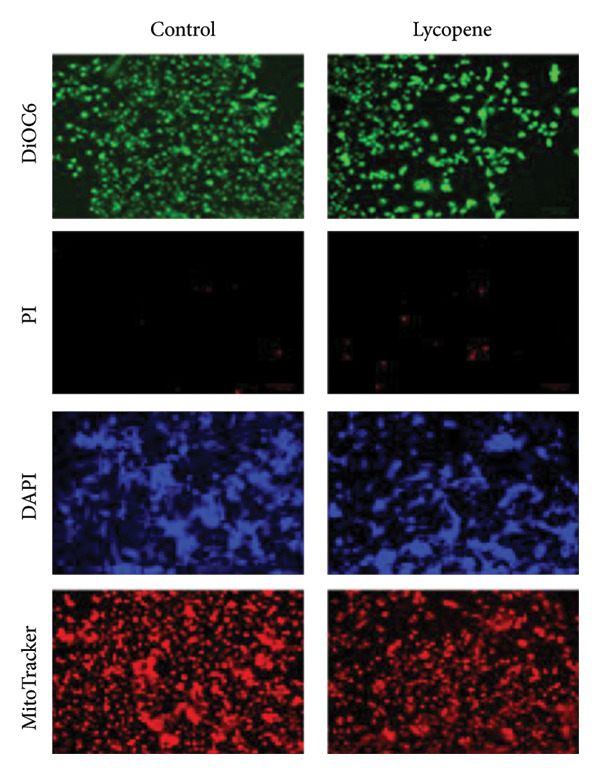
(c)

**Figure:**
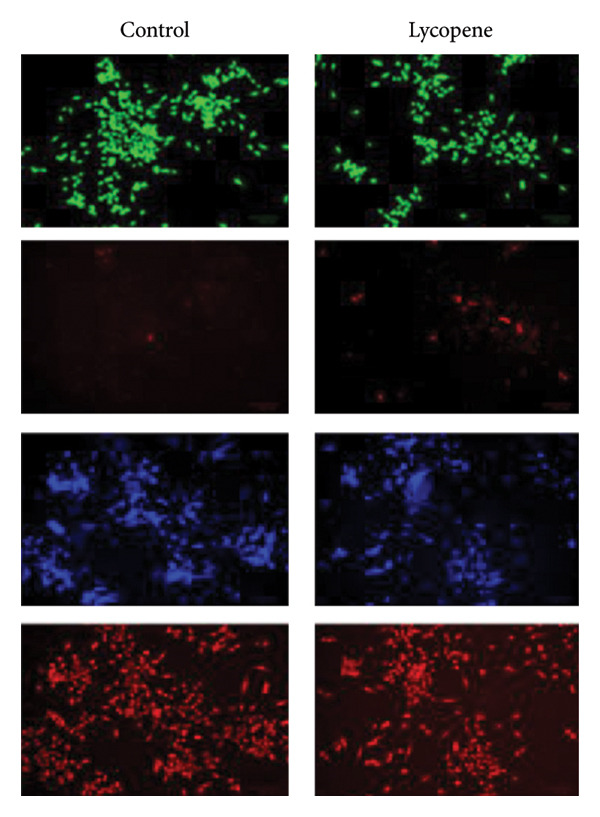
(d)

Figure 3ROS (green) and DAPI (blue) fluorescence images for all cell types: (a) control groups and (b) 50 μM lycopene treatment application for 24 h. The scale bars of the images were 100 μm.

**Figure:**
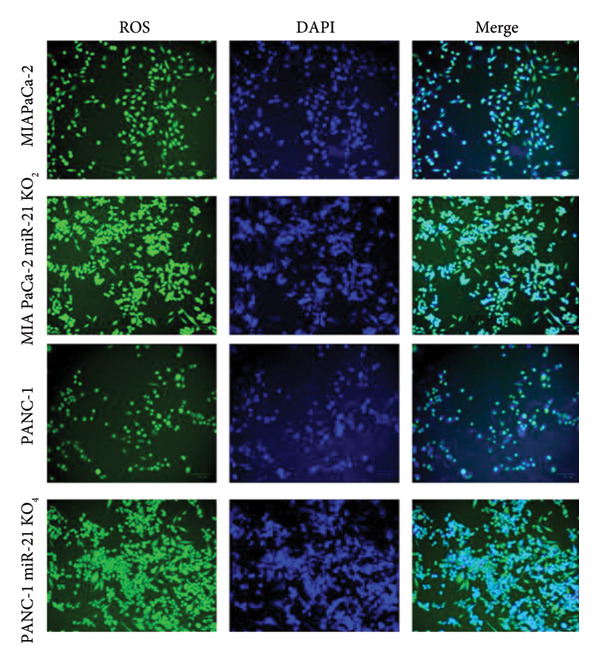
(a)

**Figure:**
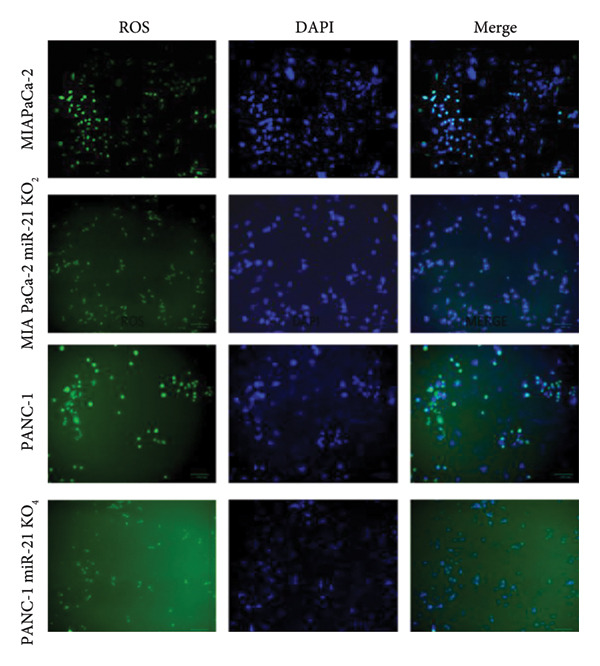
(b)

**Figure 4:**
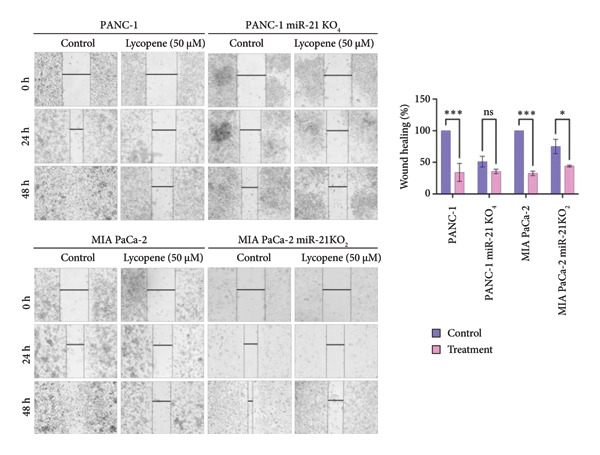
Wound‐healing assay was performed to determine the effects of lycopene treatment (50 μM) on the wound closure capacity of PANC‐1 and PANC‐1 miR‐21 KO_4_, MIA PaCa‐2, and MIA PaCa‐2 miR‐21 KO_2_ cells. Wounds were photographed at 0 h, 24 h, and 48 h. The bar graph represents relative wound healing (%) at 48 h, calculated from ImageJ measurements. Columns represented the average ± std. dev. Two‐way ANOVA and Bonferroni’s comparison test were used for statistical analysis (Panc‐1, ^∗∗∗^ *p* < 0.001; MIA PaCa‐2, ^∗∗∗^ *p* < 0.001; and MIA PaCa‐2 miR‐21 KO_2_,  ^∗^ *p* < 0.05).

To be able to understand the antiproliferative and antimigratory effects of lycopene treatment on pancreatic cancer cell lines, the wound‐healing assay was performed on PANC‐1 and PANC‐1 KO_4_, MIA PaCa‐2, and MIA PaCa‐2 KO_2_ cells (Figure 4). It was seen that lycopene treatment effectively reduced wound closure by significantly decreasing the proliferation and migration capacity of PANC‐1 and MIA PaCa‐2 cells in a time‐dependent manner (Figure 4).

**Figure 5:**
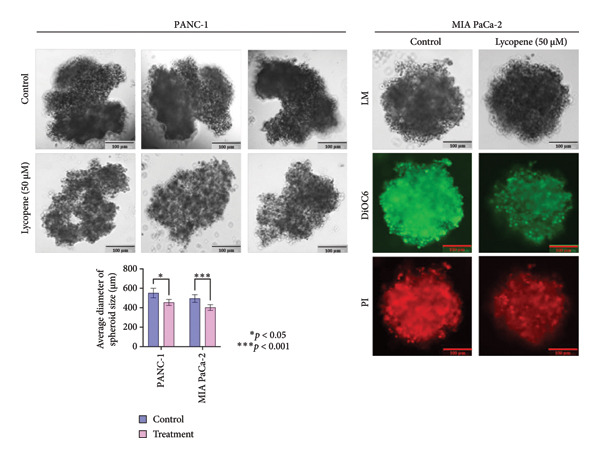
Hanging drop assay was performed on PANC‐1 and MIA PaCa‐2 cells in order to observe the effect of lycopene (50 μM) treatment on spheroid formation. Spheroids were stained with DiOC6 (green) as a viability marker and propidium iodide (PI) (red) as a necrosis marker after 72 h. Lycopene (50 μM) treatment significantly affects the spheroid size for both cell lines and the density of formed spheroids for PANC‐1 cells. The scale bar was 100 μm. Columns represented the average ± std. dev. Two‐way ANOVA and Tukey’s comparison test were used for statistical analysis (PANC‐1,  ^∗^ *p* < 0.05, and MIA PaCa‐2, ^∗∗∗^ *p* < 0.001).

The effect of lycopene treatment on tumor formation capacity for PANC‐1 and MIA PaCa‐2 cell lines was investigated by performing a hanging drop assay to obtain 3D spheroid cultures. Spheroids were observed after 72 h, as shown in Figure 5. The binding of neighboring cells to each other allows the tightening of the connection between cells, resulting in spheroid formation. It was seen that lycopene treatment affects the cell‐to‐cell adhesion capacity of PANC‐1 cells. When PANC‐1 cells were treated with lycopene, they could not compact with the decreased spheroid density and size. On the other hand, although MIA PaCa‐2 cells were able to compact after lycopene treatment, when cells were treated with lycopene, the spheroids formed were significantly smaller than nontreated cells (Figure 5).

## 4. Discussion

Lycopene is a natural pigment and a powerful antioxidant in the carotenoid family. It is found in high amounts in tomatoes and tomato‐based products, as well as in watermelons, pink grapefruits, and some other fruits and vegetables. Its potential health benefits, especially its association with cancer prevention, have made it a subject of increasing research interest. Although most lycopene studies have focused on prostate cancer, recent investigations have expanded to gastrointestinal and pancreatic malignancies.

miR‐21 has been extensively studied in the context of pancreatic cancer. miR‐21 is upregulated in pancreatic cancer cells and associated with tumor progression, metastasis, and resistance to therapy [25]. The overexpression of miR‐21 in pancreatic cancer cells promotes cell proliferation, inhibits apoptosis, enhances invasion and metastasis, and contributes to angiogenesis. These effects collectively contribute to the aggressive nature of pancreatic cancer. Recent studies indicated that miR‐21 may be involved in regulating various target genes and signaling pathways that are critical for pancreatic cancer development. For instance, miR‐21 has been shown to target tumor suppressor genes, such as PTEN and PDCD4, leading to their decreased expression and promoting cancer cell survival and proliferation [25].

Furthermore, the high levels of miR‐21 in pancreatic cancer have been associated with chemoresistance. It has been shown to target genes involved in chemotherapy response, including genes involved in DNA repair, apoptosis, and drug metabolism, thereby reducing the effectiveness of treatment. Given the significant involvement of miR‐21 in pancreatic cancer progression and therapy resistance, it is being explored as a potential therapeutic target. Researchers are investigating strategies to inhibit miR‐21, such as using antisense oligonucleotides or developing miR‐21 inhibitors, to restore normal cellular processes and enhance the effectiveness of treatment.

Lycopene is a good singlet oxygen scavenger and scavenges free radicals. It was observed that lycopene treatment leads to loss of viability among all our cell lines that were assessed with the MTT assay. It was concluded that as the time of lycopene administration increased in all cell groups, its toxicity was higher. When wild‐type and miR‐21 knockout cells were compared with each other, it was seen that lycopene was more effective in miR‐21 knockout cells. Since miR‐21 has an aggressive phenotype in cells, it is a consistent and logical result that cell survival was lower in the lycopene treatment of the miR‐21 knockout group. In the PANC‐1 KO_4_ cell line, lycopene exposure caused a time‐dependent decrease in the colony‐forming capacity of the cells. Importantly, lycopene treatment was more effective in miR‐21 knockout cells, suggesting that the absence of miR‐21 sensitizes pancreatic cancer cells to redox modulation and cytotoxic stimuli.

When the cellular ROS levels compel the cell’s antioxidant capacity, it leads to oxidative stress in the cell. This oxidative stress can modulate several signaling pathways, such as modulating cancer cell metabolism. In cancer, deregulated ROS metabolism is the hallmark of cancer cells. ROS levels in cancer can cause antiapoptotic behaviors by activating redox‐sensitive transcription factors such as NF‐κB. The study suggests that lycopene can suppress ROS‐mediated cancer cell growth, and treating with lycopene results in a significant decrease in cellular ROS levels among all cell lines. The most effective ROS control has been achieved with miR‐21 knockout cell lines. As cell viability assays, inhibition of ROS gave a more significant result in the knockout group. This is a correlated result due to miR‐21 being a related signaling pathway with drug resistance in cancer [26]. In the wound‐healing assay, it was seen that lycopene treatment effectively reduced wounds by significantly decreasing the proliferation and migration capacity of PANC‐1 and MIA PaCa‐2 cells in a time‐dependent manner.

Although lycopene treatment caused a significant decrease in cell viability and intracellular ROS levels, MitoTracker staining did not show substantial changes in mitochondrial activity. This finding may suggest that lycopene’s cytotoxic effects are not primarily mediated through mitochondrial membrane depolarization, which is the key determinant of MitoTracker dye accumulation. Instead, lycopene may exert its effects through mitochondria‐independent apoptotic or cytostatic pathways, such as modulation of NF‐κB, Akt, or p53 pathways, or through inhibition of proliferative signaling in the cytoplasm. Furthermore, MitoTracker stains mitochondria based on membrane potential, and not mitochondrial number or mass, and cells undergoing early apoptosis may still maintain membrane potential temporarily. Hence, a lack of visible change in the MitoTracker signal does not necessarily contradict the cytotoxic phenotype observed with PI and DiOC6 staining.

While these in vitro results are promising, it is essential to contextualize lycopene’s effect against current therapeutic standards. Pancreatic cancer treatment commonly involves gemcitabine or nab‐paclitaxel, which, despite being standard‐of‐care, often result in severe systemic toxicity and limited survival benefits. In contrast, Lycopene is naturally derived, well‐tolerated, and has demonstrated low toxicity and antioxidant synergism in prior preclinical models. There is also growing interest in combining Lycopene with chemotherapeutic agents to enhance efficacy while reducing adverse effects. However, Lycopene’s poor aqueous solubility, limited bioavailability, and rapid metabolism in vivo remain major barriers to clinical translation. Thus, lycopene may be best positioned as an adjuvant agent, complementing rather than replacing existing cytotoxic regimens [26, 27].

In conclusion, our study demonstrates that lycopene modulates cancer cell behavior and reduces viability, particularly in miR‐21 knockout models. These findings provide new insight into miR‐21‐related antioxidant vulnerability and support lycopene’s potential as a chemopreventive or adjunctive treatment in pancreatic cancer. While this study demonstrates that lycopene holds significant promise as a chemopreventive agent in pancreatic cancer, particularly in the context of miR‐21 suppression, further research is warranted to fully realize its therapeutic potential. Nevertheless, as a future direction, studies should explore synergistic interactions between lycopene and established chemotherapeutic agents, especially in the context of miR‐21 inhibition. To validate clinical relevance, in vivo models such as orthotopic xenografts or patient‐derived xenograft (PDX) systems should be utilized to assess pharmacodynamics, bioavailability, and tumor response. These models would also provide valuable insight into lycopene’s potential to modulate the tumor immune microenvironment or overcome drug resistance, critical challenges in the treatment of pancreatic cancer.

## Conflicts of Interest

The authors declare no conflicts of interest.

## Funding

This research is part of a TÜBİTAK 2209 undergraduate project, supported by the Scientific and Technological Research Council of Türkiye (TÜBİTAK).

## Data Availability

Data will be made available on request.
